# Immunomodulation in the repair of osteonecrosis of the femoral head: reprogramming strategies for macrophages and immune cells

**DOI:** 10.3389/fimmu.2026.1887379

**Published:** 2026-07-23

**Authors:** Longfei Han, Mingli Han, Wenyuan Hou, Guifeng Luo, Long Tian, Meixia Li, Hai Su, Mincong He, Fan Yang, Wei He, Qiushi Wei

**Affiliations:** 1 Guangzhou University of Chinese Medicine, Guangzhou, China; 2 Shenzhen Pingle Orthopedic Hospital, Shenzhen, China

**Keywords:** biomaterials, inflammation, macrophage, osteonecrosis of the femoral head, reprogramming

## Abstract

Osteonecrosis of the femoral head (ONFH) is a severe and debilitating disease that substantially affects patients’ functional capacity and daily activities. Within necrotic femoral heads, immune cells, particularly macrophages, undergo phenotypic reprogramming. This phenotypic shift in macrophages, along with their interactions with other cell types—including osteoclasts, mesenchymal stem cells, and endothelial cells—collectively mediates disease progression. However, targeted immunotherapeutic strategies remain poorly defined. In this context, the present study reviews recent literature and provides a comprehensive overview of the immunological landscape of macrophages in osteonecrosis of the femoral head (ONFH). It summarizes current knowledge on macrophage immune reprogramming, intercellular interactions with neighboring cells (such as mesenchymal stem cells, osteoclasts, and vascular endothelial cells), and the cellular composition of immune cells (including neutrophils, B cells, and T cells) in ONFH. Furthermore, it explores the potential application of biomaterials developed through various strategies for ONFH treatment. Emerging evidence indicates that the direction of macrophage polarization and their intercellular interactions with diverse cell types (e.g., mesenchymal stem cells, osteoclasts, and vascular endothelial cells), together with the infiltration of immune cells (such as neutrophils, B cells, and T cells), collectively influence the progression of ONFH. On this basis, therapeutic strategies targeting macrophage polarization and immune cell activation are proposed, with a particular focus on biomaterials possessing diverse physicochemical properties, thereby offering insights to facilitate the clinical translation of ONFH treatments.

## 1 Introduction

Osteonecrosis of the femoral head (ONFH), also known as avascular necrosis, is a debilitating condition characterized by osteocyte death resulting from impaired blood supply to the subchondral bone (1–3). Based on etiology, ONFH is classified into traumatic and non-traumatic types. The underlying pathology involves microcirculatory compromise within the subchondral bone, particularly affecting the small retinacular vessels, which ultimately leads to osteonecrosis, structural collapse, and joint degeneration (4). Direct causes include trauma (e.g., femoral head or neck fractures, hip dislocation), sickle cell disease, myeloproliferative disorders, dysbarism, and radiation exposure (5). Indirect risk factors—such as corticosteroid use, alcohol abuse, smoking, systemic lupus erythematosus, hemoglobinopathies, and hypercoagulable states (6)—contribute to bone tissue necrosis, structural remodeling, and subsequent collapse (7). Among these, corticosteroid therapy and alcohol abuse are the most prevalent risk factors for ONFH (8, 9). Reversing osteonecrosis remains a major clinical challenge. The reparative process following osteonecrosis progresses through four sequential stages: inflammation, necrosis, regeneration, and remodeling (10). In non-traumatic ONFH, which predominantly involves aseptic inflammation that impedes lesion regeneration, modulation of the immune microenvironment within bone tissue is critical for achieving disease reversal.

The relationship between immune cells (e.g., macrophages, Th17 cells, and neutrophils) and bone-related cells is intricate, and their interactions contribute to the development and progression of orthopedic diseases (11). The concept of “osteoimmunology” has recently emerged, initially emphasizing the regulatory role of T lymphocytes in osteoclast activation (12). As research into the intercellular molecular interactions and signaling pathways shared between bone and the immune system has expanded, osteoimmunology has evolved into a rapidly advancing interdisciplinary field. Maintaining a balance between the M1 and M2 macrophage phenotypes is critical. Classically activated M1 macrophages exhibit pro-inflammatory properties, whereas alternatively activated M2 macrophages exert anti-inflammatory functions. Studies have reported a significant increase in M1 macrophages in animal models of osteonecrosis of the femoral head (ONFH) (13). Macrophage-mediated pro-inflammatory responses may accelerate ONFH progression, whereas inducing the transition of macrophages from the M1 to the M2 phenotype reduces inflammatory cytokines and alleviates symptoms (14). Although the role of M1 and M2 macrophage polarization in the onset and progression of ONFH has been investigated (13), the dynamic process of bone immunity within the necrotic zone involves interactions among multiple cell types—an aspect that remains underexplored. In this review, we summarize how macrophages influence the occurrence and progression of ONFH by modulating other cell populations in the necrotic area, including bone marrow-derived mesenchymal stem cells, vascular endothelial cells, and osteoclasts. This review provides significant insight into the multifaceted pathways through which macrophages within the necrotic zone affect the progression of ONFH.

T cells mediate cellular adaptive immunity and are categorized into T helper (Th), regulatory (Treg), and cytotoxic subsets. B cells mediate humoral immunity, and evidence indicates that cytokine-mediated interactions between T and B cells play significant roles in the pathogenesis of ONFH (15, 16). Neutrophils also contribute to the immunoregulation of ONFH. The formation of neutrophil extracellular traps (NETs) is closely associated with microcirculatory disturbances that lead to ischemia in the femoral head, thereby playing a key role in the pathogenesis of ONFH (17). Moreover, NETs can directly or indirectly modulate the levels of inflammatory cytokines, thereby influencing immune cell function (18).

In conclusion, macrophage plasticity enables distinct macrophage phenotypes to assume diverse roles during the inflammatory phase, positioning these cells as central regulators of the inflammatory response in ONFH. Nevertheless, the complex regulatory networks and recent advances concerning macrophage phenotypes involved in ONFH remain insufficiently elucidated. In this context, the present review synthesizes recent literature to summarize the origins and differentiation pathways of macrophages, as well as the polarization behavior of M1 and M2 macrophages, with a particular focus on the immune reprogramming of macrophages, their intercellular interactions with mesenchymal stem cells, osteoclasts, and vascular endothelial cells, and the relationship between pathological changes—such as the extent of immune infiltration by T cells, B cells, and neutrophils in necrotic bone areas—and the progression of ONFH. Building on this foundation and drawing from the literature, this study examines preclinical therapeutic strategies for ONFH aimed at controlling inflammatory cascades and modulating M1 and M2 macrophage polarization. Furthermore, it explores biomaterials with multiple bioactive properties (including physicochemical characteristics, metallic properties, and scaffold-based delivery of active ingredients) that promote bone repair by modulating the molecular behavior of various cell types—such as enhancing osteogenic differentiation and bone formation in bone marrow mesenchymal stem cells, promoting angiogenesis, regulating macrophage polarization, and inhibiting osteoclast activation. Collectively, these findings provide insights into the immunological features of the osteonecrotic zone, in which macrophages play a dominant role, as well as into the development of promising therapeutic strategies and biomaterials for bone repair.

## 2 Roles of macrophages and other immune cells in the progression of ONFH

### 2.1 Characteristics and functions of macrophage subpopulations in orthopedic diseases

#### 2.1.1 Origins and diverse functions of macrophages

Macrophages play critical roles in inflammation and innate immunity. Upon exposure to risk factors such as mechanical stress, injury, or infection, macrophages in the blood and tissues initiate inflammatory responses through cytokine secretion and migration, which can ultimately lead to tissue damage. Macrophages are broadly categorized into two populations: tissue-resident macrophages, which originate from progenitor cells in the yolk sac, and monocyte-derived macrophages, which arise from hematopoietic stem cells in the bone marrow (19). Resident macrophages populate most tissues and contribute to the maintenance of physiological homeostasis. In contrast, inflammatory macrophages derived from circulating monocytes respond to pathological stimuli and transiently infiltrate sites of inflammation (20). Macrophages originate from monocytes (21), and diverse macrophage populations with distinct polarization states and activation markers commonly coexist *in vivo (*22). They are widely distributed and participate in a range of processes, including immunometabolism, anti-inflammatory responses, immunomodulation, hematopoiesis, maintenance of internal homeostasis, tissue injury, and remodeling. Macrophages eliminate target cells via phagocytosis and are involved in inflammatory responses, antigen presentation, and various other biological functions (23, 24). Their involvement in such diverse biological processes reflects their high plasticity and adaptability to complex microenvironments. Macrophage polarization refers to the functional activation of macrophages into two major phenotypes, defined by the expression of leukocyte differentiation antigens: classically activated M1 macrophages, which exert pro-inflammatory functions, and alternatively activated M2 macrophages, which exhibit anti-inflammatory functions (25).

#### 2.1.2 The role of macrophage polarization in bone repair

**Figure 1:**
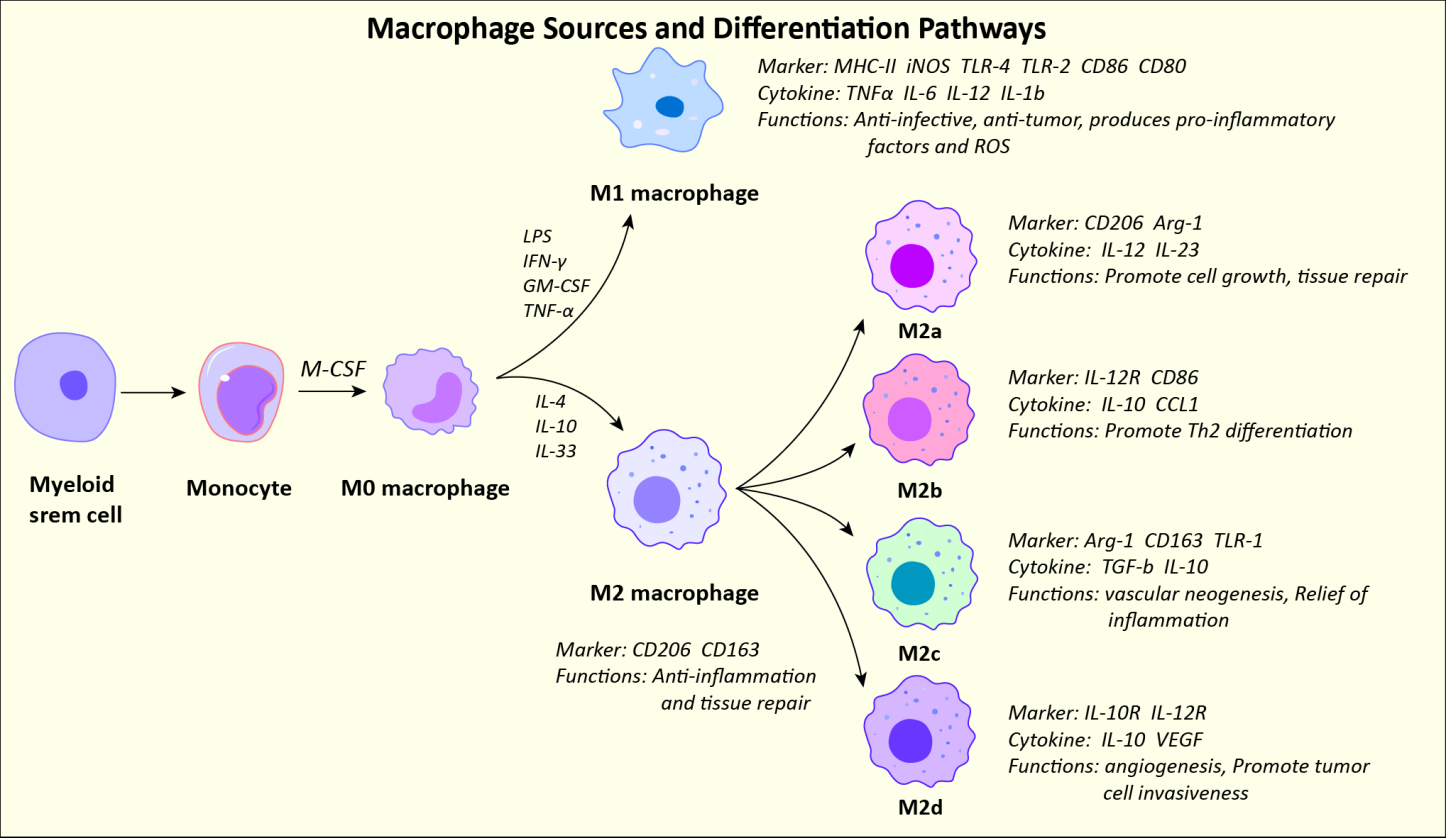
Schematic diagram of macrophage origin and differentiation.

Specifically, macrophages undergo M1 polarization upon stimulation with lipopolysaccharide (LPS) or Th1 cytokines (e.g., IFN-γ, GM-CSF, and TNF-α), whereas Th2 cytokines (e.g., IL-4, IL-10, and IL-13) induce M2 polarization. During polarization, macrophages exhibit distinct morphologies: M1 macrophages appear flat and rounded, whereas M2 macrophages assume a spindle-shaped morphology. Macrophages in these two polarized states express distinct surface markers and perform divergent functions. M1 macrophages secrete pro-inflammatory cytokines such as IL-6, IL-12, and tumor necrosis factor (TNF), and express characteristic markers including CD80, CD86, and CD16/32 (26). In contrast, M2 macrophages express high levels of arginase-1 (Arg-1), the mannose receptor (CD206), and the anti-inflammatory cytokine IL-10, as well as the chemokines CCL17 and CCL22, which help regulate and resolve inflammatory responses. M2 macrophages also play essential roles in tissue repair, angiogenesis, and bone metabolism (27). They secrete vascular endothelial growth factor (VEGF) and matrix metalloproteinases (MMPs), thereby promoting angiogenesis and tissue remodeling during the repair of osteonecrosis. Depending on the inflammatory microenvironment, M2 macrophages can be further subdivided into distinct subtypes, including M2a, M2b, M2c, and M2d (28), as illustrated in **Figure 1**. Macrophage colony-stimulating factor (M-CSF) and granulocyte-macrophage colony-stimulating factor (GM-CSF) are key regulators of macrophage phenotype and function. M-CSF, a macrophage growth factor (29), is essential for the proliferation, differentiation, and growth of M2 macrophages, as well as for tissue repair, and plays a critical role in maintaining tissue homeostasis. GM-CSF, primarily a growth factor for bone marrow progenitors (30), promotes macrophage proliferation, differentiation, and maturation. Additionally, GM-CSF influences the immune system by driving T cells to promote the generation of M1 macrophages. Collectively, both M-CSF and GM-CSF contribute significantly to macrophage proliferation, differentiation, and phenotypic switching.

### 2.2 Macrophage and immune cell reprogramming in bone repair

#### 2.2.1 Macrophage-mediated inflammatory responses in bone repair

Cellular reprogramming refers to the capacity to redefine cellular identity by altering epigenetic and transcriptional landscapes, leading to the acquisition of new morphological, molecular, and functional characteristics. These changes result in a complete reversal of cell fate and function, primarily achieved through three mechanisms: nuclear transfer, cell fusion, and forced expression of transcription factors (31). In regenerative medicine, cellular reprogramming facilitates the generation of specific cell types required for tissue repair and regeneration. Strategies for immune system reprogramming have predominantly focused on the modification of T lymphocytes. In contrast, macrophage phenotypic polarization has been implicated in the pathogenesis of various diseases, including cancer, neurodegenerative disorders, autoimmune diseases, and metabolic disorders (32). Within orthopedics, the role of macrophages in the pathogenesis of several conditions—such as osteoarthritis, rheumatoid arthritis, and ONFH—has become increasingly well defined. During bone repair, the phenotypic transition of macrophages within specific microenvironments plays a critical role in promoting the resorption of necrotic bone and the formation of new bone. In osteonecrotic conditions, bone remodeling involves the renewal of the bone matrix through two primary processes: resorption of necrotic bone by osteoclasts and new bone formation by osteoblasts (33). This process is typically accompanied by dynamic inflammatory responses. Bone reconstruction occurs in three phases: the inflammatory phase, the repair phase, and the remodeling phase (34). In the early stages of injury, acute inflammation develops around necrotic bone as a protective response to bone tissue damage, facilitating the clearance of dead cellular debris, bone fragments, and other harmful irritants through inflammatory processes (35, 36). During this phase, osteoblasts and necrotic cells at the injury site further modulate the inflammatory response by releasing endogenous stimulatory factors, including cytokines and chemokines, which recruit immune cells such as macrophages and neutrophils via the circulatory system. During the inflammatory phase, macrophages polarize toward the M1 phenotype, promoting osteoclast differentiation and activation through the secretion of pro-inflammatory cytokines, including IL-1β, IL-6, TNF-α, and IL-11. Activated macrophages and osteoclasts phagocytose and eliminate inflammatory stimuli, including bone fragments and cellular debris (37). Concurrently, pro-inflammatory factors recruit mesenchymal stem cells (MSCs) to initiate osteogenic and angiogenic processes (34, 38). Following elimination of the irritant, the inflammatory response subsides, and macrophages transition from the M1 to the M2 phenotype. M2 macrophages further enhance MSC and osteoblast differentiation and proliferation by releasing anti-inflammatory and reparative cytokines, including BMP-2, BMP-4, TGF-β, and IL-10. This transition facilitates the resolution of inflammation and initiates bone tissue regeneration at the injury site (35). Thus, the immunomodulation of macrophages represents a dynamic and critical process in the removal of necrotic bone and subsequent regenerative bone reconstruction.

#### 2.2.2 Macrophage metabolic reprogramming

Metabolism not only provides cells with the necessary energy but also plays a crucial role in maintaining the homeostasis of macrophage reprogramming (39, 40). AMP-activated protein kinase (AMPK) is a sensor that responds to energy and nutrient status and is ubiquitously expressed in eukaryotic cells (41). It promotes catabolism while simultaneously inhibiting anabolism, thereby playing a critical role in the regulation of cellular energy homeostasis. Notably, AMPK regulates various metabolic pathways and sustains energy homeostasis in T cells, macrophages, and dendritic cells (42). Positioned at the intersection of macrophage metabolism and inflammation, AMPK regulates macrophage polarization toward an anti-inflammatory (M2) phenotype through specific signaling mechanisms (43). The maintenance of M1/M2 homeostasis depends significantly on the metabolic state of the microenvironment. Previous studies have shown that pro-inflammatory M1 macrophages primarily engage in glycolytic metabolism, whereas anti-inflammatory M2 macrophages predominantly utilize fatty acid oxidation (FAO) pathways (44, 45). Thus, macrophage metabolism constitutes a critical link in the process of phenotypic reprogramming.

### 2.3 Macrophage dysregulation in the progression of osteonecrosis

#### 2.3.1 Imbalanced macrophage polarization promotes osteonecrosis progression

The pathogenesis of ONFH is multifaceted, involving factors such as intraosseous hypertension, lipid metabolism disorders, and microcirculatory disturbances (e.g., intravascular coagulation and arterial vasculitis); however, the precise underlying mechanisms remain to be elucidated. Numerous scholars suggest that the dynamic balance between bone formation and resorption is regulated by immune function, with various inflammatory cells and their cytokines playing a critical role in maintaining bone homeostasis (12). A notable characteristic of ONFH is the presence of an abnormal chronic inflammatory response, which impedes the reconstruction and repair of necrotic bone (46). Research indicates that hormone therapy induces an aberrant immune response, exacerbating vascular damage and contributing to the progression of ONFH (47, 48). Macrophage density in the ischemic necrotic region of the femoral head correlates with the underlying etiology. Studies have shown that traumatic ONFH is characterized by a lower density of macrophages in the necrotic zone, whereas hormone-induced osteonecrosis is associated with a significantly higher macrophage density in the same region (49). Alterations in macrophage dynamics are closely associated with non-traumatic osteonecrosis of the femoral head (NONFH). Research has demonstrated that macrophages and inflammatory mediators are significantly enriched in areas of necrotic repair (50), and that necrotic bone can activate macrophage-mediated inflammatory responses via Toll-like receptor 4 (TLR4) (14). Notably, the density of inflammatory cells and macrophages decreases significantly during the progression of osteonecrosis to the end stage. Furthermore, the M1/M2 ratio serves as an indicator of the proportion of pro-inflammatory macrophages. This study found that the M1/M2 ratio increased from 3 to 10 as osteonecrosis progressed toward the end stage, suggesting an enhancement of the macrophage-mediated inflammatory response. Additionally, the M1/M2 ratio in the avascular area was determined to be 12, whereas the ratio surrounding neovascularization in the repair area was approximately 0.05, indicating a lower level of inflammation in the neovascularized region. These findings suggest that during the progression of osteonecrosis, M1-type macrophages predominantly accumulate in the necrotic zone, while M2-type macrophages primarily concentrate in the repair zone, indicating spatial differences in M1/M2 ratios associated with distinct histopathological characteristics (47).

#### 2.3.2 Crosstalk between macrophages and other cells drives osteonecrosis progression

##### 2.3.2.1 Macrophages and bone marrow mesenchymal stem cells

The crosstalk between macrophages and bone marrow mesenchymal stem cells (BMSCs) contributes to the progression of ONFH. Studies have shown that the number of M1 macrophages is significantly elevated in both patients and mouse models of steroid-induced osteonecrosis of the femoral head (SIONFH). Moreover, exosomes derived from glucocorticoid-treated M1 macrophages regulate the adipogenic differentiation of BMSCs, with miR-1a-3p playing a crucial role by targeting CEBPZ. Inhibition of miR-1a-3p expression significantly impairs the adipogenic differentiation of BMSCs, thereby slowing the progression of SIONFH (51). In contrast, exosomes derived from M2 macrophages promote the proliferation and osteogenic differentiation of BMSCs while suppressing the expression of inflammatory factors, suggesting that M2 macrophages may inhibit ONFH progression through exosomal mechanisms (52). Bioinformatics analyses have further revealed that ONFH is closely associated with an imbalance among neutrophils, monocytes, and M2 macrophages, as well as with the activation and resting states of dendritic cells (53). Therefore, targeting macrophages and modulating their polarization states to achieve macrophage reprogramming may represent an effective therapeutic strategy for addressing the immune imbalance underlying ONFH (54). As anti-inflammatory macrophages, M2 macrophages exert bone-repairing effects by suppressing the inflammatory responses of neighboring cells. Research by Le et al. (55) indicates that miR-122 in M2 macrophages promotes the osteogenic differentiation of BMSCs, enhances cell viability, and stimulates the expression of osteogenesis-related genes (RUNX2, BMP2, OPN, and ALP). These findings suggest that harnessing the anti-inflammatory potential of M2 macrophages represents a promising therapeutic strategy.

##### 2.3.2.2 Macrophages and osteoclasts

Osteoclasts, the sole cells responsible for bone resorption, are derived from monocyte/macrophage precursor cells upon stimulation by RANKL. Studies have confirmed that within the necrotic areas of ONFH, the expression of cathepsin K (CTSK), a marker of osteoclasts, is upregulated, and the number of tartrate-resistant acid phosphatase (TRAP)-positive cells is increased (56). Furthermore, plasma levels of osteoprotegerin (OPG) and receptor activator of nuclear factor κB ligand (RANKL) in patients with ONFH are significantly higher than those in healthy controls, indicating that osteoclasts are in a state of excessive activation during the progression of ONFH (57). One hypothesis suggests that the surge in osteoclast activity leads to excessive bone resorption, outpacing bone formation, thereby directly weakening the subchondral structure of the affected femoral head and leading to collapse. Consequently, interventions targeting osteoclasts hold therapeutic promise. Macrophages, as cells capable of producing inflammatory factors, can promote bone resorption by influencing osteoclast differentiation. Kazuya Uehara et al. (58) investigated the interaction between macrophages and osteoclasts using necrotic bone fragments in *in vitro* experiments. The results indicated that necrotic bone fragments promote the release of soluble factors by macrophages, which in turn further activate the ERK–Bcl-xL axis in osteoclasts and enhance their anti-apoptotic capacity, thereby promoting bone resorption. This finding also explains why necrotic bone within the femoral head contributes to a cascade of amplification and a vicious cycle in the progression of necrosis. Senescent cells secrete the senescence-associated secretory phenotype (SASP), which includes growth factors, cytokines, and chemokines. These factors act in concert to create a toxic microenvironment (59), thereby disrupting the normal function of neighboring cells. Studies have confirmed that senescence in cells of the monocyte/macrophage lineage contributes to the inflammatory response by promoting excessive osteoclast activation via SASP factors, thereby exacerbating the progression of ONFH. However, treatment with senolytic agents (dasatinib and quercetin) can eliminate senescent cells within the femoral head, reducing the number of osteoclasts in mice with ONFH and thereby slowing the progression of osteonecrosis (60). These findings suggest that macrophages in osteonecrosis may also promote osteoclast activation through factors secreted by senescent macrophages.

##### 2.3.2.3 Macrophages and endothelial cells

Capillaries in bone tissue are mainly classified into three subtypes: H-type, L-type, and E-type (61). H-type vessels are primarily confined to the epiphyseal ends and the region beneath the periosteum, with specific markers including CD31 and EMCN (62). The prevailing view is that H-type vessels are closely associated with ONFH. Using single-cell RNA sequencing, researchers have found that the number of endothelial cells and H-type endothelial cells within the femoral head decreases with increasing severity of necrosis in ONFH. Furthermore, in necrotic samples, angiogenic capacity is reduced and accompanied by elevated levels of inflammation (63).

Endothelial-mesenchymal transition (EndoMT) refers to the process by which endothelial cells migrate out of the endothelial layer while losing their endothelial characteristics and acquiring mesenchymal traits (64). This process affects endothelial function and contributes to the pathological progression of microcirculatory dysfunction in ONFH. Researchers have investigated the relationship between M2 macrophages and the phenotypes of neutrophils and endothelial cells via the exosome pathway. Their findings indicate that M2 macrophages in the necrotic bone region of ONFH can reduce the formation of neutrophil extracellular traps (NETs) through the enrichment of miR-93-5p in their exosomes, while also improving the angiogenic capacity of endothelial cells (65). This suggests that M2 macrophages within necrotic bone participate in the bone repair process through the exosome pathway by promoting phenotypic changes in endothelial cells. Collectively, the pathological changes in ONFH do not represent isolated alterations in cellular function but are mediated through extensive intercellular interactions within the necrotic zone.

**Figure 2:**
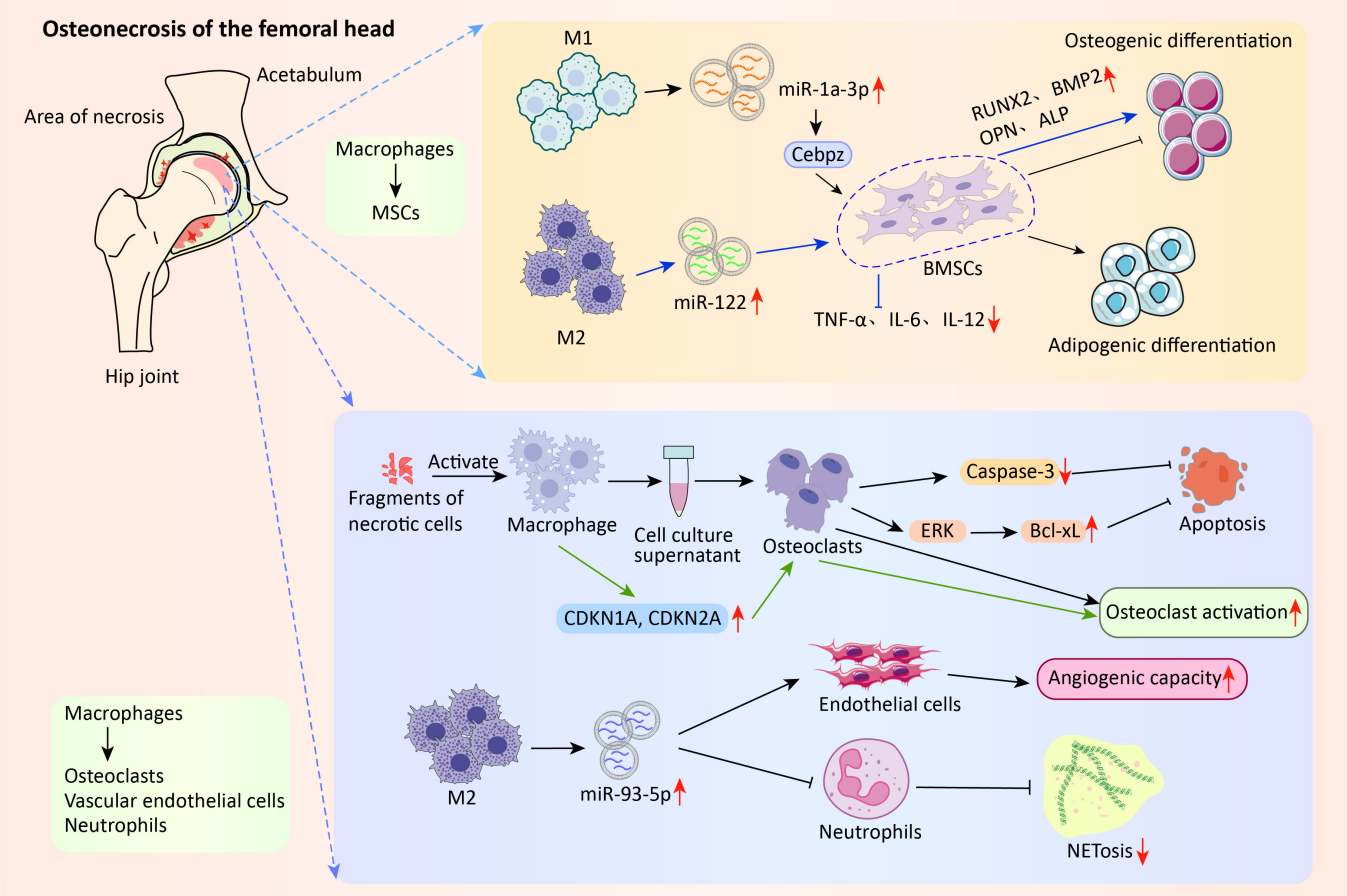
Cellular interactions between macrophages and osteoclasts, mesenchymal stem cells, endothelial cells, and neutrophils in the necrotic bone of ONFH.

In conclusion, macrophages not only influence the progression of ONFH through their own cellular reprogramming but also, through interactions with mesenchymal stem cells, osteoclasts, and vascular endothelial cells, alter the phenotypic and biological characteristics of these other cell types, thereby collectively shaping the pathological course of ONFH, as summarized in **Table 1** and **Figure 2**.

**Table 1:** Interactions between macrophages and other cell types in the necrotic zone of ONFH.

Cell types	Researcher	Specific cellular interactions	Key findings	Cite
Macrophages and Mesenchymal Stem Cells	1. Guoping Le et al. 2. Na Yuan et al.	M2 Macrophages and Bone Marrow Mesenchymal Stem Cells	1.In M2 macrophages, miR-122 promotes the osteogenic differentiation of BMSCs, enhances cell viability, and upregulates the expression of osteogenesis-related genes, including RUNX2, BMP2, OPN, and ALP. 2.Exosomes derived from M2 macrophages promote the proliferation and osteogenic differentiation of BMSCs while suppressing the expression of pro-inflammatory mediators.	(52, 55)
	Yi Zheng et al.	Adipose-Derived Mesenchymal Stem Cells (ADSCs) and M1 Macrophages	Exosomes derived from ADSCs mitigate the progression of osteonecrosis by inhibiting the NF-κB/NLRP3/IL-1β axis, thereby reducing M1 macrophage apoptosis and the release of pro-inflammatory cytokines.	(66)
	Liangjie Bai et al.	Macrophages and BMSCs	The bio-scaffold described in this article promotes M2 polarization of macrophages and mitigates inflammation by scavenging excess reactive oxygen species (ROS), thereby establishing a favorable immune microenvironment for the osteogenic differentiation of BMSCs.	(67)
	1. Duan P et al. 2. Yannan Cheng et al.	M1 macrophages and BMSCs	1. M1 macrophages and BMSCs are co-localized within bone tissue. Exosomes released by M1 macrophages inhibit the osteogenic differentiation of BMSCs and, via miR-1a-3p, further target Cebpz to promote adipogenic differentiation. 2. Conditioned medium from M1 macrophages activates the NF-κB pathway through TNF-α, thereby inducing apoptosis in BMSCs.	(51, 68)
	Gang Tian et al.	bilical Cord Mesenchymal Stem Cells (hUC-MSCs) and Macrophages	hUC-MSCs attenuate osteocyte apoptosis through the suppression of M1 macrophage polarization.	(69)
Macrophages and Osteoclasts	Kazuya Uehara et al.	Macrophages and Osteoclasts	Conditioned medium from macrophages pre-treated with necrotic bone tissue enhances osteoclast survival by activating ERK signaling and upregulating Bcl-xL expression.	(58)
	Peng Wang et al.	Bone Marrow-Derived Macrophages (BMDMs) and Osteoclasts	Single-cell RNA sequencing reveals that patients with ONFH exhibit an increased number of osteoclasts and elevated expression of macrophage senescence-associated genes, including CDKN1A and CDKN2A. Macrophage senescence may promote excessive osteoclast activation and contribute to ONFH progression; therefore, the elimination of senescent cells represents a promising therapeutic strategy.	(60)
	Ryohei Kozutsumi et al.	Macrophages and Osteoclasts	Macrophage exhaustion promotes the shift toward an M1 phenotype by increasing the proportion of CD38^+^ M1 macrophages and decreasing that of CD163^+^ M2 macrophages, while also reducing osteoclast numbers, thereby facilitating the progression of osteonecrosis.	(70)
Macrophages and Endothelial Cells	Guanzhi Liu et al.	M2 Macrophages and Endothelial Cells	Exosomes derived from M2 macrophages, enriched with miR-93-5p, mediate the reduction of neutrophil extracellular trap formation and the enhancement of endothelial cell angiogenic capacity.	(65)

#### 2.3.3 Immune cells in the progression of osteonecrosis

##### 2.3.3.1 Immune microenvironment dysregulation in ONFH

The skeletal system is intricately linked to the immune system (71). The bone marrow houses hematopoietic stem cells and mature immune cells, including B cells, macrophages, and a limited population of T cells; these systems interact through shared regulatory cytokines, chemokines, receptors, signaling molecules, and transcription factors (72). Among these components, RANKL serves as a pivotal molecule that establishes a clear connection between the skeletal and immune systems. A growing body of evidence suggests that the dynamic balance between bone formation and resorption is modulated by inflammatory activity, with various inflammatory cells and their cytokines playing crucial roles in maintaining bone homeostasis (12). A distinctive feature of ONFH is the presence of a chronic inflammatory response, in which necrotic bone acts as a significant pathological factor that exacerbates inflammation (54). To elucidate the pathological characteristics of the necrotic zone in idiopathic ONFH, researchers performed comprehensive transcriptomic sequencing of femoral head bone tissue. The results revealed a close association between ONFH and elevated inflammatory levels, along with significant downregulation of genes involved in angiogenesis, cell migration and adhesion, and osteoblast differentiation (73). These findings underscore the critical role of immune homeostasis in the pathogenesis of ONFH.

##### 2.3.3.2 T cells and B cells

An imbalance in T-cell and B-cell subsets contributes to the pathophysiological processes underlying ONFH. CD4^+^ and CD8^+^ T cells represent major T-cell subsets. Naïve CD4^+^ T cells differentiate into various helper T cell subsets—including Th1, Th2, Th9, Th17, Th22, and regulatory T cells (Tregs)—as well as follicular helper T cells, depending on environmental stimuli (74). Among these, Th1 cells produce IFN-γ, which regulates cellular immunity, while Th2 cells produce IL-4 and IL-5, thereby mediating humoral immunity. Th17 cells promote osteoclastogenesis by upregulating RANKL expression, which enhances bone resorption, whereas Treg cells (CD4^+^, CD25^+^, Foxp3^+^) maintain bone homeostasis by inhibiting osteoclast differentiation (75). CD8^+^ T cells play an integral role in modulating immune responses and function as cytotoxic T lymphocytes that mediate cellular lysis (76). In patients with non-traumatic ONFH, the numbers of CD3^+^ T cells, suppressor T cells (CD3^+^ CD8^+^), and B-1 cells (CD5^+^ CD19^+^) were significantly elevated compared with normal controls. Notably, T-cell counts correlated with ARCO stage, as evidenced by a significantly lower percentage of T lymphocytes in patients with ARCO stage IV compared with those with stages II and III, and a significantly lower percentage of suppressor T lymphocytes in patients with CJFH type L3 compared with those with types L1 and L2. Among ONFH patients with different etiologies, the total numbers of lymphocytes and suppressor T cells were significantly lower in those with alcohol-associated ONFH than in those with idiopathic ONFH (15). Animal studies have demonstrated significantly lower levels of Foxp3^+^ CD4^+^ regulatory T cells and significantly higher levels of RANKL^+^ T cells in the bone marrow of the femoral head in rats with hormone-induced ONFH (77). Collectively, these findings suggest that T cells and B cells play a significant role in the pathogenesis of ONFH.

T cells regulate bone remodeling by secreting cytokines in an autocrine or paracrine manner. Activated T lymphocytes produce receptor activator of nuclear factor κB ligand (RANKL), thereby promoting osteoclastogenesis (78). This, in turn, increases the RANKL/OPG ratio, contributing to enhanced bone resorption and subsequent bone loss (79). Recent studies have indicated that Th17 cells play a crucial role in autoimmune inflammatory diseases and bone remodeling through the production of IL-17 (80). The inflammatory subpopulation of Th17 cells functions as a subset of osteoclast-inducing T helper cells, linking T cell activation to bone resorption. Hee Yeon Won et al. (81) observed that CD4^+^ T cells stimulated by inflammation promote IL-17 secretion, which subsequently induces RANKL-mediated osteoclastogenesis. Normal bone remodeling relies on a dynamic balance between osteoclast-mediated bone resorption and osteoblast-mediated bone formation. An imbalance between these two cell types contributes significantly to the pathogenesis and progression of ONFH.

##### 2.3.3.3 Neutrophils

Growing evidence suggests that inflammation and immune dysregulation are associated with ONFH. Neutrophils, as the first responders in host antimicrobial defense and in aseptic inflammation (such as in ONFH), play a crucial role in both health and disease. Studies utilizing peripheral blood assays have investigated biomarkers in patients with ONFH. Results indicate that levels of C-reactive protein (CRP), the neutrophil-to-lymphocyte ratio (NLR), monocyte percentage, and platelet counts are significantly elevated in ONFH patients (82, 83), and these markers may serve as tools for early screening and detection of the disease. Jiang et al. (18) used correlation analysis on peripheral blood samples and found that the levels and percentages of monocyte-macrophages, neutrophils, eosinophils, and basophils differed significantly between ONFH patients and normal controls, suggesting that both systemic and local inflammatory mechanisms contribute to disease development in ONFH. Consequently, peripheral blood neutrophil counts may be used as an adjunctive diagnostic tool for ONFH; however, direct evidence demonstrating the molecular mechanisms by which neutrophils contribute to the onset and progression of ONFH remains lacking and warrants further investigation.

Neutrophils play a crucial role in bone immunomodulation, with one of their most significant functional phenotypes being NETosis and the subsequent release of neutrophil extracellular traps (NETs), which contain histones, granzymes, and nuclear components (65, 84). NETs are extracellular networks composed of DNA and neutrophil cytoplasmic granules released by activated neutrophils (85). Although NETs are essential for innate immunity, their excessive formation and delayed degradation can lead to their accumulation in tissues, resulting in cellular damage, autoimmune diseases, and microcirculatory disorders due to their prothrombotic properties (86). Research on the relationship between ONFH and NETs remains limited. To investigate this association, Nonokawa et al. (87) assessed the presence of NETs in the femoral heads of patients with ONFH and hip osteoarthritis (HOA) using immunofluorescence staining. The results revealed a significant increase in NETs within the small blood vessels surrounding the femoral head in ONFH patients, and demonstrated that intravenously injected NETs can enter the tissues around the femoral head and potentially induce femoral head ischemia. Similarly, Hodaka Ogawa et al. investigated the effects of methylprednisolone pulse therapy on the development of ONFH in a mouse model of systemic lupus erythematosus (SLE). The findings indicated that methylprednisolone pulse therapy induces the formation of NETs in the microvasculature surrounding the femoral head, which may lead to microcirculatory disturbances and promote femoral head ischemia (17).

## 3 Treatment strategies for osteonecrosis

### 3.1 Macrophages, immune cells, and the repair of osteonecrosis

Initial studies addressing the role of macrophages and other immune cells in bone repair primarily focused on fracture healing. Specifically, excessive accumulation of activated neutrophils at the fracture site has been implicated in impaired fracture healing (88). Furthermore, neutrophils can recruit substantial numbers of macrophages by secreting inflammatory and chemotactic mediators such as IL-6 and CCL2. Recently, a population of bone-resident macrophages termed osteomacs has been identified and shown to collaborate with neutrophils in regulating bone repair (89). Macrophages eliminate the provisional fibrin matrix and necrotic cells through phagocytosis. Concurrently, they promote the recruitment of fibroblasts, mesenchymal stem cells (MSCs), and bone progenitor cells by secreting various inflammatory and chemotactic mediators, including tumor necrosis factor α (TNF-α), IL-1β, IL-6, and CCL2 (90). In addition, macrophages orchestrate the proliferation, differentiation, and extracellular matrix production of MSCs and bone progenitor cells through the secretion of growth factors. Members of the transforming growth factor β (TGF-β) family—particularly TGF-β1, TGF-β2, and TGF-β3—along with bone morphogenetic proteins (BMPs), vascular endothelial growth factor (VEGF), platelet-derived growth factor (PDGF), and fibroblast growth factor-2 (FGF-2), are also involved in this process (91).

### 3.2 Suppression of inflammation facilitates bone repair through macrophage polarization and immune cell activation

In the SIONFH animal model, infiltration of neutrophils and macrophages is observed in osteonecrotic tissues. Furthermore, necrotic bone can stimulate macrophages to mediate the inflammatory response through activation of Toll-like receptor 4 (TLR4) and upregulation of its downstream transcription factors, including nuclear factor-κB (NF-κB) and monocyte chemotactic protein-1 (MCP-1) (14). During osteonecrosis repair, macrophages transition from the M1 to the M2 phenotype, facilitating immune cell reprogramming. Consequently, the acute inflammatory response is resolved and replaced by a mesenchymal stem cell (MSC)-rich environment and repair tissue abundant in extracellular collagen matrix and neovascularization (92). Thus, controlling inflammation is critical during necrotic bone repair, as successful clearance of inflammatory stimuli is accompanied by the production of anti-inflammatory and reparative cytokines that eliminate the inflammatory environment and re-establish tissue homeostasis. However, if the inflammatory stimulus persists and the bone repair process is altered, the condition may progress to a chronic inflammatory state. This aberrant inflammatory response exerts sustained tissue-destructive effects and disrupts immune homeostasis through the persistent secretion of low levels of inflammatory cytokines, which is detrimental to the repair of osteonecrosis. The macrophage reprogramming strategy operates along the monocyte-macrophage (M1/M2 differentiation)-osteoclast pathway, with dynamic crosstalk occurring alongside other pathways, such as the MSC-osteoclast pathway. These interactions represent two of the most significant aspects of necrotic bone repair (33).

### 3.3 Suppresses M1 polarization of macrophages

M1 macrophages exert detrimental effects on the progression of osteonecrosis. Studies have shown a significant increase in the number of M1 macrophages within the femoral heads of patients with SIONFH and in corresponding mouse models. *In vitro* experiments have further demonstrated that conditioned medium derived from M1 macrophages induces apoptosis in bone marrow mesenchymal stem cells (BMSCs) and MLO-Y-4 osteocyte-like cells. This effect is mediated by TNF-α present in the conditioned medium, which activates the NF-κB pathway. Conversely, macrophage depletion significantly reverses the detrimental effects attributed to M1 macrophages (68). Furthermore, Kim et al. demonstrated that flushing cellular debris and inflammatory proteins from the necrotic femoral head using necrotic zone lavage in piglets with ONFH led to increased bone formation by reducing the numbers of osteoclasts and macrophages, suggesting that elimination of pro-inflammatory macrophages facilitates ONFH healing (93). Astragaloside IV (AS-IV), an extract derived from Astragalus membranaceus, has been shown to enhance immune function (94, 95). Research indicates that AS-IV promotes the transition of macrophages from the M1 to the M2 phenotype, enhances osteoblast survival, alleviates arthritic symptoms, and reduces levels of inflammatory cytokines, thereby slowing ONFH progression (96). Interleukin-6 (IL-6) is a key pro-inflammatory cytokine that plays a significant role in initiating acute inflammatory responses and sustaining chronic inflammatory states. Studies have reported significantly elevated IL-6 levels in the synovial fluid of patients with Legg-Calvé-Perthes disease (LCPD). In a piglet model of ONFH, treatment with tocilizumab, a monoclonal antibody targeting the IL-6 receptor, markedly ameliorated the pathological manifestations of hip synovitis. Tocilizumab treatment also reduced the number of synovial macrophages and osteoclasts on the surface of necrotic bone, thereby enhancing bone healing (97). In addition, animal studies revealed that IL-6 knockout did not affect the extent of osteonecrosis; however, the necrotic epiphyses exhibited significantly increased vascularization. Furthermore, IL-6 knockout significantly increased osteoblast numbers, as well as the bone formation rate (BFR) and mineral apposition rate (MAR) on the necrotic side. These findings suggest that IL-6 plays a critical role in osteonecrosis repair by regulating macrophage levels and influencing vascularization in necrotic areas (98).

Obesity constitutes a significant risk factor for osteonecrosis and is associated with a chronic inflammatory state (99). Animal studies have shown that a high-fat diet (HFD) is associated with increased severity of osteonecrosis and elevated macrophage IL-6 expression in SIONFH mice. In contrast, IL-6 knockdown or macrophage depletion mitigated the effects of HFD on osteonecrosis, suggesting that anti-IL-6 therapy or depletion of pro-inflammatory macrophages may facilitate osteonecrotic repair (100). Curcumin (C_21_H_20_;O_6_), the principal active component of turmeric, possesses notable anti-inflammatory properties. *In vivo* experiments demonstrated that curcumin attenuates M1 macrophage infiltration in the femoral head of SIONFH mice. Additionally, *in vitro* studies indicated that curcumin inhibits M1 macrophage polarization via the JAK1/2-STAT1 pathway, thereby suppressing inflammatory responses and osteoclast apoptosis (101). Hesperidin (HSN), a citrus flavonoid predominantly derived from citrus fruits, exhibits significant anti-inflammatory and antioxidant potential (102). Research has revealed that HSN reduces the expression of matrix metalloproteinases (MMPs) in synovial fibroblasts and inhibits M1 macrophage polarization through the PI3K/AKT signaling pathway (103).

### 3.4 Promote M2 polarization of macrophages

Alpha-2-macroglobulin (A2M) is a multifunctional protein involved in metabolite clearance, protease inhibition, and the regulation of cellular physiological processes. Shanhong Fang et al. (104) reported that A2M activates the Keap1/Nrf2 pathway to regulate oxidative stress and apoptosis while promoting M2 macrophage proliferation, thereby alleviating the symptoms of ONFH. Myricetin (β-glucoside) is widely used in the treatment of orthopedic diseases (105). Research has shown that, compared with normal bone tissue, the M1/M2 macrophage ratio is significantly elevated in necrotic bone associated with SIONFH. Additionally, the levels of TLR4, MYD88, and NF-κB p65 are markedly higher than those observed in patients with hip osteoarthritis. *In vivo* experiments demonstrated that myricetin attenuates osteonecrosis and the destruction of trabecular bone structure in a dose-dependent manner, accompanied by the promotion of M1-to-M2 macrophage conversion and downregulation of TLR4, MYD88, and NF-κB p65 expression (106). Bone marrow adipose tissue serves as a significant source of cytokine and chemokine production. Several studies have investigated the phagocytosis of necrotic adipose tissue by macrophages and the resulting phenotypic transformation, indicating that macrophages infiltrate the repair tissue during ONFH healing. Immunofluorescence co-localization experiments demonstrated that macrophages phagocytosed necrotic adipocytes. Findings from both *in vivo* and *in vitro* experiments revealed that phagocytosis of adipocytes decreased the expression of M1 macrophage markers (e.g., IL-1β and iNOS), accompanied by a concomitant increase in M2 marker expression (e.g., Arg1). These results suggest that macrophage phagocytosis of adipose tissue induces macrophage polarization, offering a novel approach for macrophage-based immunomodulatory therapy (107). Exosomes are extracellular vesicles that transport proteins and RNAs involved in intercellular communication and signaling (108). It has been demonstrated that chondrocyte-derived exosomes can promote M2 macrophage polarization by delivering miR-214-3p to modulate the ATF7/TLR4 axis, while also promoting angiogenesis via the RUNX1/VEGFA axis (109).

**Figure 3:**
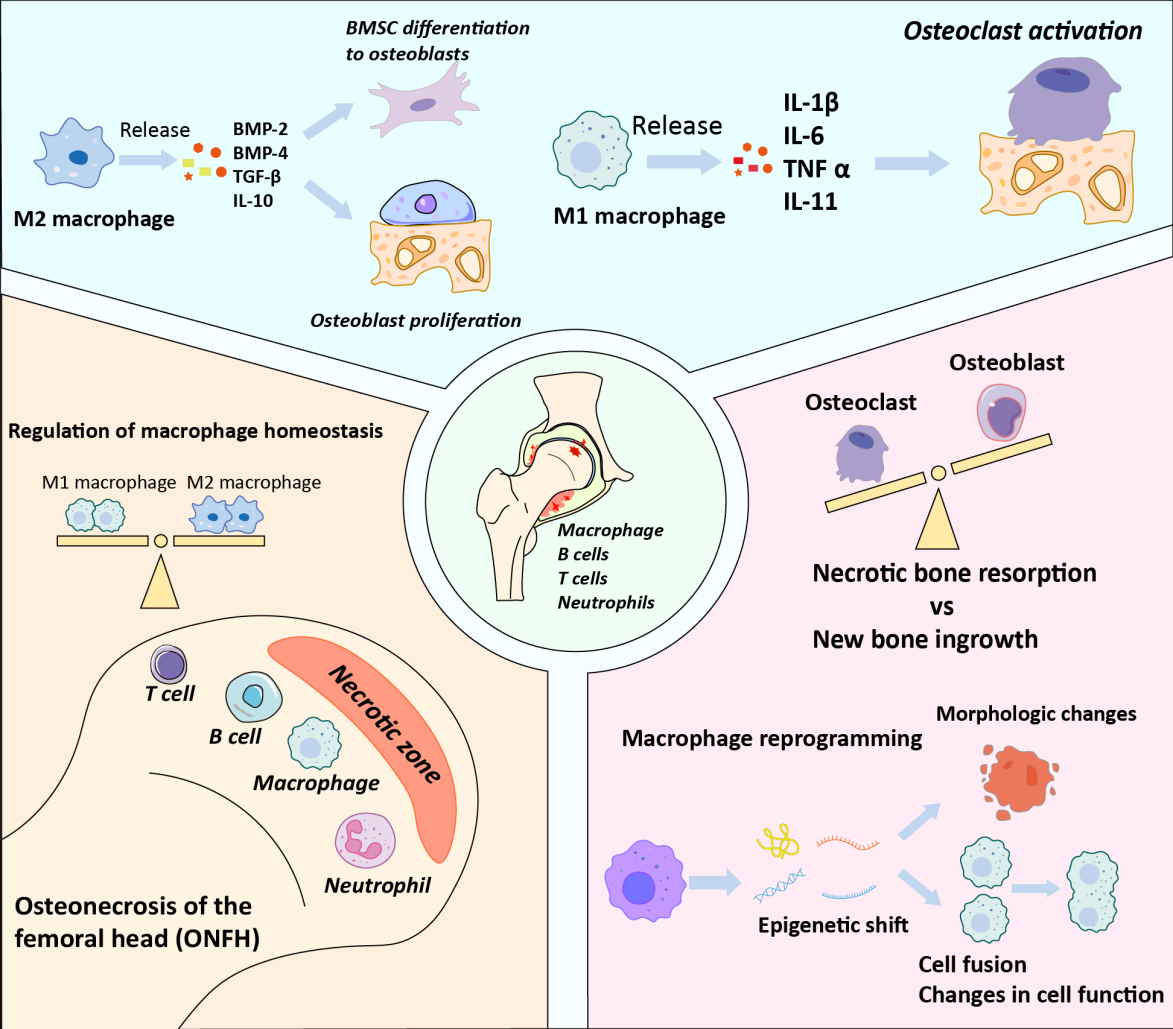
The role of immune cells, including macrophages, in immune reprogramming in ONFH.

In summary, therapeutic strategies that involve reprogramming macrophage polarization play a beneficial role in preserving inflammatory homeostasis within the necrotic bone in ONFH, as illustrated in **Figure 3**.

### 3.5 Biomaterials for modulating immune cell infiltration and macrophage reprogramming

#### 3.5.1 Biomaterials engineered with tailored physical or chemical properties

Significant progress has been made in the development of medical biomaterials capable of inhibiting and reversing the pathological processes associated with osteonecrosis of the femoral head (ONFH) by modulating immune infiltration through physical and/or chemical approaches. In the context of bone repair, bioelectrical signals derived from living bone—including piezoelectricity, pyroelectricity, and ferroelectricity—are critical regulators of bone metabolic activity. Therefore, the development of smart coatings that respond to environmental changes and subsequently enhance cell viability and bone defect regeneration holds considerable practical value (110, 111). Barium titanate (BaTiO_3_) ceramics exhibit excellent biocompatibility, bioactivity, and osteogenic capacity. Studies have shown that the piezoelectric potential of BaTiO_3_ promotes apatite deposition, cell differentiation, and bone formation. A porous BaTiO_3_ coating on Ti6Al4V, fabricated via three−dimensional (3D) printing, not only stimulates the osteogenic differentiation of mesenchymal stem cells (MSCs) and the angiogenic function of endothelial cells but also regulates the immune microenvironment and enhances bone regeneration by inhibiting the MAPK/JNK inflammatory pathway and promoting macrophage polarization toward the M2 phenotype (112). In another study, a thermosensitive hydrogel carrier was developed using poly(lactic−co−glycolic acid) (PLGA)−polyethylene glycol (PEG)−PLGA (113). This hydrogel system generates microcurrents in piezoelectric nanofibers upon ultrasound stimulation, thereby enhancing the physiological activity of stem cells through electrical stimulation while simultaneously mitigating the detrimental effects of Th17 cells on stem cells via chemical mechanisms. Consequently, synergistic modulation of immune infiltration in ONFH through combined physical and chemical strategies is likely to significantly improve therapeutic efficacy. Precise regulation of macrophage polarization is critical in the management of chronic inflammation associated with ONFH. Drawing inspiration from innate immunity, a recent study employed microfluidic technology to fabricate immune cell−mobilizing hydrogel microspheres. These microspheres were constructed using a mixture of streptavidin−grafted hyaluronic acid methacrylate (HAMA−SA) and chondroitin sulfate methacrylate (ChSMA), and were loaded with chemokines, macrophage antibodies, and engineered extracellular vesicles via both covalent and non−covalent attachment (110). *In vitro* experiments demonstrated a remarkable ability of these microspheres to recruit, capture, and reprogram macrophages, effectively converting pro−inflammatory macrophages into anti−inflammatory phenotypes. Furthermore, *in vivo* experiments indicated that these microspheres attenuated synovial inflammatory responses, highlighting their significant research value for the repair of necrotic areas in ONFH (114).

Magnetic field−assisted bone regeneration has emerged as a promising strategy in tissue engineering and has recently garnered increasing research interest (115). Magnetic nanoparticles (MNPs), owing to their unique responsiveness to magnetic fields, hold significant potential for application in bone tissue engineering (116). Zhou et al. (117) developed a magnetically responsive biphasic calcium phosphate (HBCP/Fe_3_O_4_) scaffold with a hollow tubular whisker structure embedded with nanoparticles. *In vitro* experiments demonstrated its excellent bone regeneration capacity, as evidenced by the promotion of osteogenic differentiation of bone marrow−derived mesenchymal stem cells (BMSCs), enhancement of angiogenesis in human umbilical vein endothelial cells (HUVECs), and induction of M2 polarization in RAW 264.7 macrophages. Mechanistically, under magnetic stimulation, the scaffold activates Piezo1 to mediate Ca²^+^ influx, which in turn triggers the BMP−2/Smad signaling pathway, thereby promoting the osteogenic differentiation of BMSCs.

#### 3.5.2 Metallic materials with bone-regenerative properties

The application of metallic materials in the treatment of bone and joint diseases shows considerable promise. Scaffold materials, typically composed of bioceramics or metallic components, exhibit mechanical properties closely resembling those of natural bone and have been widely used in orthopedic implants (118, 119). Furthermore, these scaffolds can modulate the immune response by modifying their microscopic morphology to enhance wettability and by carrying bioactive substances, thereby promoting bone repair through regulation of the immune microenvironment (120, 121). However, their inherent plasticity may be limited, making it difficult to fill irregular bone defects. Consequently, injectable hydrogels have gained increasing attention in orthopedics (122–124). Recent studies have shown that biomimetic multifunctional scaffolds loaded with desferrioxamine (DFO), zinc−modified metal–organic framework 818 (Zn−MOF−818), gelatin methacryloyl (GelMA) hydrogel, and demineralized bone matrix (DBM) can inhibit oxidative stress, promote angiogenesis, and regulate immune responses. This immunomodulation is particularly evidenced by the promotion of macrophage polarization toward the M2 phenotype and the attenuation of inflammatory responses (67). Metal ions not only serve as essential trace elements for metabolic balance and tissue regeneration but have also been recently shown to possess biological activities integral to bone regeneration and immunomodulation (125–128). In one study, an injectable thiolated hyaluronic acid hydrogel combined with lithium−doped nanohydroxyapatite (Li−nHA@Gel) enabled sustained and prolonged release of lithium ions, which enhanced M2 macrophage polarization via activation of the JAK1/STAT6/STAT3 signaling pathway. This mechanism mediates osteogenic and angiogenic processes by modulating the immune microenvironment (129).

## 4 Discussion

ONFH is a challenging and debilitating condition characterized by various underlying causes that disrupt blood supply to the femoral head, ultimately leading to cell death within the bone tissue. If left untreated, this condition can progress to femoral head collapse, joint destruction, and eventually to hip osteoarthritis (HOA) (130). Clinical data reveal significant macrophage infiltration in the osteonecrotic areas of patients with ONFH when compared to adjacent bone tissue (47). As key components of the immune system, macrophages play a critical regulatory role in chronic inflammatory responses (35). The presence of necrotic bone, under the constant influence of chronic inflammation, impairs the processes of bone reconstruction and repair, leading to the exacerbation of ONFH. Consequently, various strategies are employed to target macrophages and modulate the immune microenvironment within the necrotic area to enhance the bone tissue repair response.

Bone immune homeostasis is a complex and dynamic process involving multiple inter-regulating mechanisms (131). Historically, studies on bone immunity have predominantly focused on the interactions among T lymphocytes, B lymphocytes, and neutrophils (15, 132). However, recent research has increasingly highlighted that macrophage polarization is a critical factor in the regulation of bone metabolism and represents a promising target for the treatment of disorders related to bone immune homeostasis, particularly in conditions such as ONFH (131). The complexity of macrophage phenotypes contributes significantly to the regulation of bone immune homeostasis. M1-type macrophages, as pro-inflammatory cells, promote bone formation during the acute inflammatory phase; however, during the chronic inflammatory phase, M1-type macrophages facilitate bone resorption, adversely affecting bone formation and leading to an imbalance in bone homeostasis. In contrast, M2-type macrophages release anti-inflammatory factors in the later stages of inflammation to mitigate local inflammatory responses. Additionally, they induce osteoblast differentiation, which enhances bone mineralization, promotes bone formation, and aids in the repair of necrotic bone (88). Therefore, the modulation of macrophage polarization to maintain bone immune homeostasis may serve as an effective therapeutic strategy to promote necrotic bone repair in ONFH. In addition to regulating macrophage polarization, the use of small−molecule anti−inflammatory drugs, traditional Chinese herbal medicines, and nanomaterials with multiple biological activities to suppress the inflammatory responses of macrophages and other immune cells should be regarded as the most fundamental therapeutic strategy. This approach not only controls the inflammatory cascade in macrophages but also modulates the biological functions of mesenchymal stem cells (MSCs), osteoclasts, and vascular endothelial cells via macrophage−mediated signaling, thereby promoting bone repair in ONFH. However, our analysis indicates that although multiple cell types are present within the femoral head (63)—including MSCs, osteoblasts, endothelial cells, monocytes, T cells, B cells, natural killer (NK) cells, fibroblasts, macrophages, chondrocytes, osteoclasts, and pericytes—current studies have primarily focused on the cellular interactions between macrophages and MSCs, endothelial cells, or osteoclasts. To fully elucidate the role of macrophages in ONFH, future research should be directed toward investigating their interactions with other cell types, such as osteoblasts, fibroblasts, chondrocytes, T cells, and B cells.

In summary, recent studies have elucidated that macrophage-mediated inflammatory homeostasis is a significant mechanism underlying the progression of ONFH pathology. However, the roles of other immune cell types (e.g., T-lymphocytes, B-lymphocytes, dendritic cells, and neutrophils) in the pathological stages of ONFH remain poorly understood (77, 87). Given the heterogeneity and diversity of osteoblasts and various immune cells, transcriptomic analyses of pathological tissues in ONFH are anticipated to be instrumental in uncovering the clues related to immune reprogramming during the progression of the condition. Among these analyses, single-cell sequencing and spatial transcriptome sequencing can facilitate spatial whole transcriptome imaging at single-cell resolution, thereby providing unprecedented insights into cell-specific spatial functions and disease mechanisms (133, 134). This approach is expected to lead to breakthroughs in the study of the bone immune network in ONFH. Furthermore, further research is needed on materials that possess multiple biological functions and adequate biocompatibility to facilitate the clinical translation and application of ONFH therapies.

## 5 Conclusion

An imbalance in the immune microenvironment exacerbates the progression of osteonecrosis of the femoral head (ONFH). The underlying mechanisms include macrophage phenotypic switching, immune and metabolic reprogramming, intercellular crosstalk, and inflammatory infiltration of immune cells, which collectively intensify the inflammatory cascade within necrotic bone. Specifically, M1 macrophages exhibit enhanced glycolytic metabolism and exert pro−inflammatory effects, whereas M2 macrophages show increased fatty acid oxidation and exert anti−inflammatory effects. Macrophages interact with mesenchymal stem cells (MSCs), osteoclasts, and vascular endothelial cells, thereby influencing the progression of ONFH through multiple biological functions. These include modulating the osteogenic differentiation of MSCs, the resorptive capacity and activation status of osteoclasts, and the angiogenic capacity and mesenchymal transition of vascular endothelial cells. Furthermore, T−cell− and B−cell−mediated inflammatory infiltration can activate the RANKL pathway to promote bone resorption, while the formation of neutrophil extracellular traps (NETs) also contributes to the inflammatory cascade and disease progression in ONFH.

In terms of therapeutic strategies, leveraging the functional differences among macrophage subtypes—such as using anti−inflammatory agents or traditional Chinese herbal medicines—can reduce the inflammatory response, promote the phenotypic switch of macrophages from M1 to M2, and regulate macrophage metabolic reprogramming. These effects in turn influence the cellular functions of other cell types (MSCs, osteoclasts, and vascular endothelial cells), ultimately promoting bone repair following necrosis. Moreover, the rapid advancement of biomaterial development technologies in recent years has enabled the fabrication of metal−based biomaterials and scaffolds with excellent biocompatibility, microcurrent−generating capacity, magnetic responsiveness, and bioactivity. Their superior material properties and multi−cell−targeting biofunctional capabilities are being increasingly investigated. It is anticipated that future research will lead to the development of novel materials focused on immune reprogramming of macrophages and other immune cells, ultimately facilitating the clinical translation and application of ONFH treatment modalities.
